# Distribution of Ki-67 values within HER2 & ER/PgR expression variants of ductal breast cancers as a potential link between IHC features and breast cancer biology

**DOI:** 10.1186/s12885-017-3212-x

**Published:** 2017-03-29

**Authors:** Sven Kurbel, Branko Dmitrović, Ksenija Marjanović, Damir Vrbanec, Antonije Juretić

**Affiliations:** 1 Osijek Medical Faculty, Cara Hadrijana 10/E HR - 31000, Osijek, EU Croatia; 2 Department of Pathology and Forensic Medicine, Osijek University Hospital, Osijek, Croatia; 3 Department of Oncology, Zagreb University Hospital, Zagreb, Croatia

**Keywords:** Breast cancer, Immunohistochemistry, Cancer phenotypes, Ki-67

## Abstract

**Background:**

Unexpected differences in Ki-67 values among HER2 & ER/PgR defined subgroups were found. This study aims to detect possible subdivisions beyond the conventional breast cancer types.

**Methods:**

One thousand one hundred eighty consecutive patients with invasive ductal breast carcinoma were included and distributed in 16 subgroups (four HER2 phenotypes (0+, 1+, 2+ and 3+) times four ER/PgR phenotypes). Complex distributions of Ki-67 values were tested by expectation maximization (EM) clustering.

**Results:**

Pooled Ki67 values of all patients showed the presence of three EM clusters (defined as LMA-low mitotic activity, IMA-intermediate mitotic activity and HMA-high mitotic activity) with expected mean Ki-67 values of 1.17%, 40.45% and 77.79%, respectively. Only ER-PgR- tumors significantly dispersed in three clusters (29.75% tumors in LMA, 46.95% in IMA and 23.30% in the HMA cluster), while almost no detected HMA tumors were of ER + PgR+ or ER + PgR- phenotypes.

Among 799 ER + PgR+ patients distribution in clusters was HER2 dependent (*p* = 0.000243), due to increased number of IMA HER2 3+ tumors on the expense of LMA HER2 3+ tumors (52 IMA out of 162 HER2 3+ patients versus113 IMA out of 637 HER2 < 3+ patients). This was not found among ER + PgR- patients (*p* = 0.186968).

Among ER-PgR- patients, HER2 overexpression also increased number of IMA tumor, but by reducing the number of HMA tumors (*p* < 0.000001). Here, difference between HER2 absent (0+) and HER2 3+ patients was evident (10 HMA out of 125 HER2 3+ patients versus 42 HMA out of 103 HER2 0+ patients).

**Conclusions:**

Results suggest that distributions of breast cancers in three clusters of mitotic activity depend on different mechanisms for ER + PgR+ and ER negative tumors. Although HER2 overexpression increases number of IMA tumors in both settings, in the former it is done by reducing number of LMA tumors, while in the latter it reduces the number of HMA tumors. Mitotic activity of ER + PgR- tumors seems unrelated to the HER2 status, possibly as an indicator that ER dysfunctionality in cancers that lack PgR expression. Among ER negative tumors, the absence of HER2 (0+) might be as important as the HER2 overexpression.

**Electronic supplementary material:**

The online version of this article (doi:10.1186/s12885-017-3212-x) contains supplementary material, which is available to authorized users.

## Background

Despite many advances in cancer therapy, a majority of all drugs are of variable effectiveness in patients with a certain cancer type. In rare occasions (i.e. HER2 overexpressed breast cancer), a limited subgroup of patients has been recognized as requiring a special type of treatment, developed for their cancer variant. In many other situations, the standard therapy is applied according to the contemporary clinical guidelines. From a clinical perspective, evidence-based decisions on what type of therapy are to be used for a certain patient remain a challenging task despite development of new drugs.

In most cancer patients, contemporary stratification is based on tumor tissue morphology and is not directly related to the tumor biology, or treatment outcomes. This means th at any well established cancer type or subtype can contain several subgroups of patients whose outcome might have been improved if they were recognized as a specific subgroup and thus differently treated. A new systematic approach to the patient stratification according to tumor biology features found at the time of diagnosis is needed to improve our results in treating common cancer types. One of several possibilities is to distribute new cancer patients in subgroups based on tumor phenotype features previously validated as predictors of tumor biology and/or treatment outcomes. Clinical and histologic phenotype features linked to tumor biology might lead to new targeted therapies for certain patient subgroups, in hope of achieving better treatment outcomes.

In the diagnostic evaluation of breast cancer, estrogen receptor (ER), progesterone receptor (PgR), human epidermal growth factor receptor 2 (HER2) and Ki-67 are routinely used for the classification of breast tumors into distinct subtypes [1, 2].

The prevailing contemporary classification of breast tumors recognizes five basic immunohistochemical phenotypes: Luminal A, Luminal B1 and Luminal B2 are the three breast cancer types with positive ER or PgR expression. Among breast cancers that are both ER and PgR negative, two separate types are recognized, the triple-negative and pure HER2 tumors. The former tumors have normal HER2 expression (from 0+ to 2+), while the latter show HER2 overexpression (3+).

It was proposed by the multistep model for breast carcinogenesis suggests that invasive carcinoma arises via a series of intermediate hyperplastic lesions through various grades of atypia to in situ and invasive carcinomas [3]. This model thus assumes that there must be a continuous phenotypic range of breast lesions that leads to invasive ductal cancers instead of separate mechanisms of occurrence for the five distinct breast cancer types.

Based on the report from the Clinical Cancer Registry Regensburg in Bavaria, Germany, among 4480 patients with non metastatic breast cancers, these immunohistochemical results divided tumors in Luminal A (found in 48.4% patients), Luminal B (24.8% patients), HER2-like (17.8% patients) and Basal-like (found in 9.0% patients) [2]. In another report, among 267 patients with invasive breast carcinomas, 44.9% of tumors were Luminal B type, 21.7% Luminal A tumors, 18.7% triple-negative and 14.6% of pure HER2 type [4].

Breast cancer types are important in making therapeutic decisions. The presence of ER and PgR on tumor cells at the time of surgery guides adjuvant therapy [5], as an important predictor of both prognosis and hormone dependency. It was reported that rare negative ER/PgR positive breast cancers are biologically different from ER positive/PgR positive tumors and have a poor clinical outcome [6]. For instance, significant differences in histologic grade (*p* < 0.001) and PgR expression (*p* < 0.001) were reported between the Luminal A and B types, leading to the conclusion that different management guidelines should be considered for these two breast cancer types [4]. It was also reported that accurate classification of breast cancer patients as Luminal A, or as Luminal B is important for determining effective adjuvant treatment of ER positive and HER2 not over-expressed tumors [7].

Results from a detailed analysis of histopathological data of 1180 patients with invasive ductal breast cancer are here presented. All patients have been treated in a single regional medical center. Immunohistochemical features of primary breast tumors were analyzed according to their Ki-67 value, as a marker of mitotic activity.

**Fig. 1:**
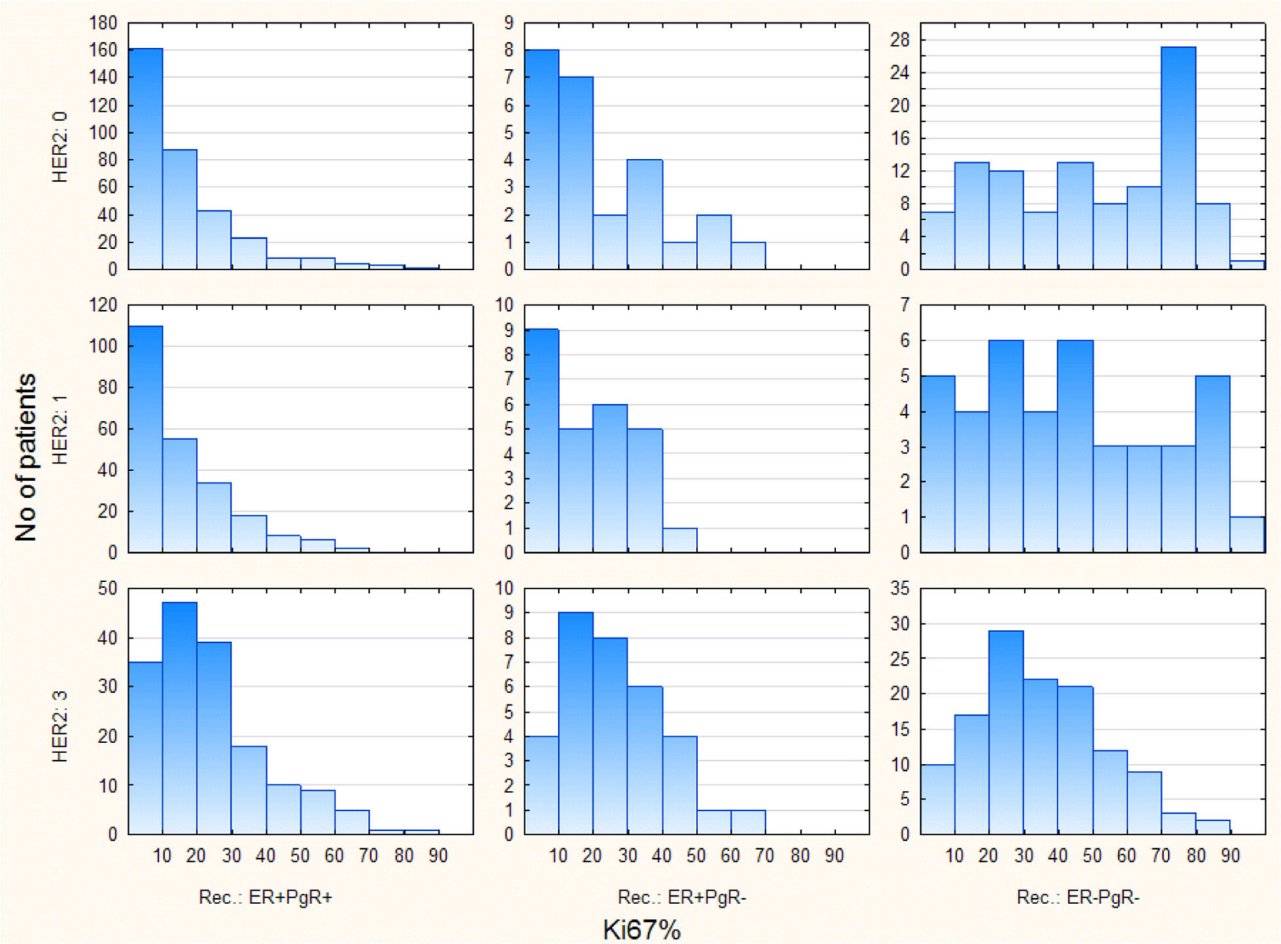
Histograms of Ki-67 values in groups of breast cancer patients accordingly to their immunohistochemical cancer phenotype

This study was inspired by the distribution of Ki67 values regarding the HER2 expression status and ER/PgR phenotype (shown in Fig. 1). Differences in ranges and trends of Ki-67 values among the three common ER/PgR phenotypes seem self-evident, so this paper is aimed at detecting whether differences in tumor Ki-67 values among subgroups of patients are caused by the existence of further subdivisions of tumors beyond usual breast cancer types.

## Methods

### Patients

In this study 1180 consecutive invasive ductal breast cancer patients (any stage) were included. All patients were diagnosed and treated in Osijek Clinical Hospital from the period January 2004 to December 2012. We have used a single institution set of breast cancer patients that has already been assembled as a part of a research project financed by the Croatian Ministry of Science (219–2,192,382-2426). Before grant submission to Croatian Ministry of Science and Education, collecting of breast cancer data was approved by the Ethical Committee of Osijek Medical Faculty, as compliant with the Helsinki Declaration. These same patients’ data were used for testing two other breast cancer models [8, 9] and the results of these testings were published elsewhere [10, 11].

All of the specimens were excisional biopsies or mastectomy specimens. Tumor grades were determined using the Bloom and Richardson scheme [12–14].

### Immunohistochemistry

All IHC slides were coded and independently evaluated by two pathologists, who are also the coauthors of this paper. They have used the ImageJ program tools (https://imagej.nih.gov/ij/) when needed. Each immunostained slide was evaluated for the presence of ER and PgR expression, HER2 protein overexpression, and Ki–67 proliferation activity. Immunohistochemical staining was done by the standard avidin-biotin method (DAKO LSAB®2 System, HRP) using 4 μm sections from representative paraffin blocks. Nuclear staining with anti-ER, PgR and Ki-67 antibodies was also done and the percentage of positive cells per 500 tumor cells was calculated. Tumor cells were considered positive for HER2 protein over-expression when greater than 10% of the cells showed strong membrane staining (equivalent to a score of 3+ in the DakoCytomation HercepTest). An HER2 2+ result was considered overexpressed only if confirmed by chromogene in situ hybridization for gene amplification. Hormone receptors were reviewed and accepted as negative if 100% of cells lacked nuclear immunostaining.

From our previous pilot study, we have noticed for the Ki-67 values that the two independently estimated values were usually less than 7% apart, so in all cases when the difference was <6% we have used the arithmetic mean of these two estimates as the final value. In less than one fifth of patients, with the ki-67 gap >5%, two new independent estimations were done. The lowest and the highest value were discarded and the arithmetic mean of the remaining two values was used.

#### Breast cancer types based on IHC features

In our first paper [10] we have used the 14% threshold in separating Luminal A and Luminal B1 breast cancers. In preparing the second paper [11], one of the main objection of reviewers was that the threshold should be 20%, based on the St. Gallen 2013 conclusion: ".. The Panel noted that standardized cut-offs for Ki-67 have not been established and laboratory specific values should be used, but the majority of the Panel voted that a threshold of >20% was clearly indicative of ‘high' Ki-67 status" [15]. Beside that, the same conclusions state: “.. The majority of the Panel accepted that a useful surrogate definition of Luminal A-like as distinct from Luminal B-like disease could be made using a combination of ER, PgR and Ki-67, without requiring molecular diagnostics” [15].

Based on the cited reference and to IHC results, tumors of our patients were divided into following five groups: Luminal A (ER+ and/or PgR+, HER2-negative, Ki-67 < =20%), Luminal B1 (ER+ and/or PgR+, HER2-negative, Ki-67 > 20%), Luminal B2 (ER+ and/or PgR+, HER2-overexpressed, any Ki-67), HER2 (ER–, PgR–, HER2-overexpressed), and triple-negative (ER–, PgR–, HER2-negative).

### Statistical analysis

Collected data were organized in a spreadsheet by StatSoft, Inc. (2011) STATISTICA (data analysis software system), version 10. www.statsoft.com.

As shown in Table 1 the usual distribution of breast cancers was based on HER2 expression, low or high Ki67 values and combinations of ER and PgR presence, thus resulting in 16 subgroups (four HER2 variants (0+, 1+, 2+ and 3+) times four ER/PgR phenotypes).

**Table 1:** Distribution of breast cancer patients according to the immunohistochemical cancer phenotype

ER/PgR expression	HER2 phenotypes		Breast cancer type	Number of patients		Total
	Binary classification	semiquantitative expression		Ki-67 < =20%	Ki-67 > 20%	
ER + PgR+	“negative”	0+	*Luminal A/B1*	248	90	338
		1+		165	68	233
		2+		44	19	63
	“overexpressed”	3+	*Luminal B2*	82	83	165
Total of patients with ER & PgR positive tumors				539	260	799
ER + PgR-	“negative”	0+	*Luminal A/B1*	15	10	25
		1+		14	12	26
		2+		4	3	7
	“overexpressed”	3+	*Luminal B2*	13	20	33
Total of patients with ER positive & PgR negative tumors				46	45	91
ER-PgR+	“negative”	0+	*Luminal A/B1*	1	3	4
		1+		3	2	5
		2+		2	0	2
	“overexpressed”	3+	*Luminal B2*	0	0	0
Total of patients with ER negative & PgR positive tumors				6	5	11
ER-PgR-	“negative”	0+	*triple-negative*	20	86	106
		1+		9	31	40
		2+		2	6	8
	“overexpressed”	3+	*pure HER2*	27	98	125
Total of patients with ER & PgR negative tumors				58	221	279
Total of all patients				649	531	1180

Out of 16 subgroups in Table 1 ER-PgR+ subgroups had too few patients to be used in statistical tests (only 11 patients), so they were excluded from further statistic tests. Further more, out of the remaining 12 subgroups (four with ER + PgR+, four with ER + PgR- and four with ER-PgR- tumors), histograms of Ki-67 distributions were made in Fig. 1 only for HER2 subgroups 0+, 1+ and 3+. The three omitted HER2 2+ cancer subgroups were not suitable for histogram comparison, due to low number of patients.

Complex distributions shown in Fig. 1 suggested that more than one cluster of patients might be present in each subgroup. Possible existence of clusters within a single phenotypic subgroup was tested by applying the method of expectation maximization (EM) clustering [16] to the original Ki-67 data of ER + PgR+, ER + PgR- and ER-PgR- breast cancers (in total 12 subgroups). The v-fold cross-validation algorithm for automatically determining the number of clusters in the data (provided by the Statistica program) was applied during the clustering. The EM algorithm of clustering approximates the observed distributions of values by a mixture of distributions in different clusters.

We have done a two-stage EM clustering. The first stage is done within the described subgroups and it suggested that all subgroup clusters belong to only three clusters present in the whole set of patients. In the second stage, all data were pooled together to verify presence of these three overall clusters that were used in further analysis.

## Results

In this study 1180 consecutive patients with invasive ductal breast cancers (regardless of stages) were included. All patients were diagnosed and treated in Osijek Clinical Hospital from January 2004 to December 2012.

### Distribution of KI-67 values regarding ER/PgR and status of HER2 expression

Distribution of Ki-67 values among the 16 proposed phenotypic subgroups are shown in Table 1. Among them, 11 out of 1180 patients (0.93%) showed the rarest ER-PgR+ cancer phenotype, so in following tables these 11 patients were excluded, thus leaving 12 subgroups with 1169 patients.

Figure 1 shows discrepancies between distributions of Ki67 values among the remaining nine subgroups of patients regarding their ER/PgR phenotype and HER2 expression (0+, 1+ or 3+, HER2 2+ tumors were omitted due to low incidences). These data were validated by Kruskal-Wallis tests:

- Among the ER + PgR+ tumors, Ki-67 values were higher in HER2 3+ cancer than in tumors with low HER2 expression (1+), or without any expression (HER2 absent) (*p* < 0.0001).
- Among the ER + PgR- tumors, no difference in Ki67 values, depending on the HER2 was found (*p* = 0.3175).
- Particularly interesting were ER-PgR- tumors (in the bottom row of Fig. 1). The highest levels of Ki-67 values are found in tumors without expression of HER2 (HER2 absent). The presence of HER2 reduced KI-67 values slightly and this downslope holds for the whole sequence of HER2 absent to HER2 3+. The difference between the cancers without HER2 (HER2 absent) and cancers overexpressing HER2 (HER2 3+), was statistically significant (*p* = 0.0003).

In short, if we compare cancers positive for ER and PgR, where HER2 expression increases Ki67 values, with the ER-PgR- cancers, were HER2 expression decreases otherwise very high KI-67 values, these unexpected differences obviously required further examinations. A plausible interpretation is that even in these narrow subgroups of breast cancers, unexpected distributions of Ki67 values might result from further subgroup divisions.

### The first stage EM clustering within subgroups of ER/PgR and HER2 phenotypes

**Fig. 2:**
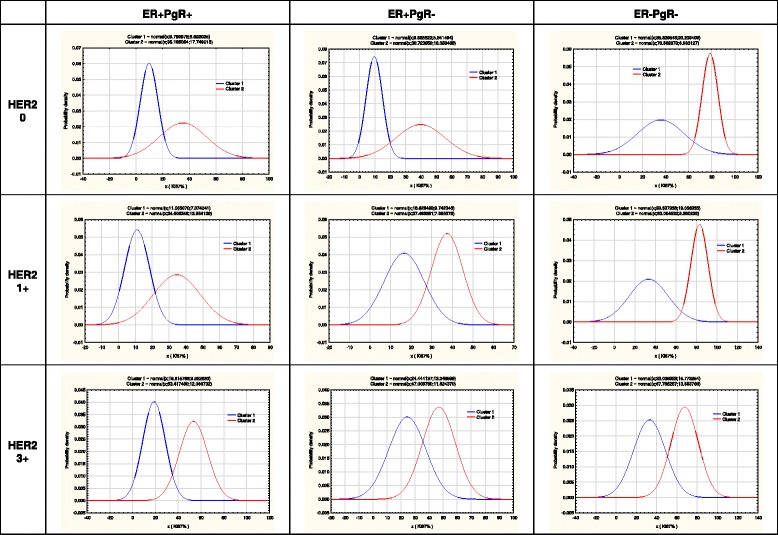
Cluster distribution within nine subgroups of breast cancer patients (shown as histograms in Fig. 1) accordingly to their ER/PgR status and HER2 expression. The first stage of EM clustering detected two clusters of patients in each subgroups (marked here as clusters 1&2). In all subgroups one cluster was of intermediate Ki-67 value (labeled IMA in Table 2), while the other showed either low (LMA in Table 2) or high values (HMA in Table 2)

Table 2 shows results of the first stage EM clustering for the analyzed subgroups. Figure 2 shows distributions of EM clusters within nine subgroups analogous to the histogram setting in Fig. 1. Despite our expectations, the v-fold cross-validation algorithm detected only two clusters of patients in each subgroup:

- In ER positive tumors, dominant clusters consisted of patients with low Ki-67 values (columns labeled LMA for Low Mitotic Activity, with mean values from 10 to 16% in Table 2.). In two ER+ and HER2 3+ subgroups, the LMA analogous clusters showed mean Ki67 values from 19 to 26%, suggesting that HER2 overexpression increases Ki67 values of tumors with low mitotic activity.
- In all HER2 absent (0+), HER2 1+ and HER2 2+ subgroups, one cluster contains patients whose tumors show intermediate Ki-67 values (near 40% are mean KI-67 values,), here defined as the IMA clusters (from Intermediate Mitotic Activity)
- In ER positive tumors, the share of IMA clusters declines with HER2 expression (among PgR+ cancers: from 25% of HER2 absent to 15% in HER2 3+; among PgR- cancers: from 40% in HER2 absent to 6.1% in HER2 3+ cancers).
- In two ER+ HER2 3+ subgroups, the intermediate range clusters shows mean Ki67 values 55 to 60%, suggesting that among these tumors HER2 overexpression increased Ki67 values and reduced share of IMA tumors.
- Among ER negative tumors HER2 expression did not boost mitotic rates of dominant IMA clusters (30 to 35%), but it reduced the share of the cluster with high Ki67 values (high mitotic activity - HMA), from 40% in HER2 absent tumors to 11.2 in HER2 3+ cancers, resulting in overall lower Ki-67 values among the pure HER2 tumors.

**Table 2:** Detected EM clusters of Ki-67 values within subgroups of breast cancer patients defined by certain immunohistochemical phenotypes (LMA - low mitotic activity; IMA - intermediate mitotic activity; HMA - high mitotic activity). These are the results of the first stage of EM clustering

ER/PgR phenotypes			Breast cancer HER2 status							
			0+		1+		2+		3+	
ER + PgR+	*Cancer types*		*Luminal A/B1*						*Luminal B2*	
	EM clusters		LMA	IMA	LMA	IMA	LMA	IMA	LMA	IMA
	Patients	n	255	83	187	46	52	11	139	26
		*%*	*75.4*	*24.6*	*80.3*	*19.7*	*82.5*	*17.5*	*84.2*	*15.0*
	Mean Ki-67%		9.5	39.1	11.3	39.6	13.5	38.6	19.2	56.6
	St.dev. of Ki-67		6.1	15.2	7.2	11.1	6.9	9.5	10.1	10.3
ER + PgR-	*Cancer types*		*Luminal A/B1*						*Luminal B2*	
	EM clusters		LMA	IMA	LMA	IMA	n/a		LMA	IMA
	Patients	n	15	10	20	6	7		31	2
		*%*	*60.0*	*40.0*	*76.9*	*23.1*	*n/a*		*93.9*	*6.1*
	Mean Ki-67%		9.7	42.4	15.5	39.0			25.4	60.0
	St.dev. of Ki-67		5.4	14.8	8.6	6.0			13.3	7.1
ER-PgR-	*Cancer types*		*triple-negative*						*pure HER2*	
	EM clusters		IMA	HMA	IMA	HMA	n/a		IMA	HMA
	Patients	n	63	43	29	11	8		111	14
		*%*	*59.4*	*40.6*	*72.5*	*27.5*	*n/a*		*88.8*	*11.2*
	Mean Ki-67%		34.0	79.0	32.3	82.7			32.4	73.2
	St.dev. of Ki-67		18.3	6.4	18.1	8.5			14.7	8.7

### The second stage EM clustering of the pooled data set

The above results of EM clustering in various subgroups suggest that in all three analyzed ER/PgR phenotypes, some patients had breast tumors that do not overexpress HER2 and have similar intermediate mitotic activity independent of the presence of ER and PgR (all IMA clusters). On the other hand, clusters of low mitotic activity (LMA) were present only in ER positive cancers (both in PgR+ and PgR- cancers). Their share was slightly reduced in subgroups with HER2 expression (1+ to 3+), suggesting that these tumors of low mitotic activity were hormone driven and thus less EGFR/HER2 dependent. Among ER-PgR- tumors, cancers of high mitotic activity formed the HMA clusters, more common in variants poor in HER2 expression (HER2 0+ and 1+).

**Fig. 3:**
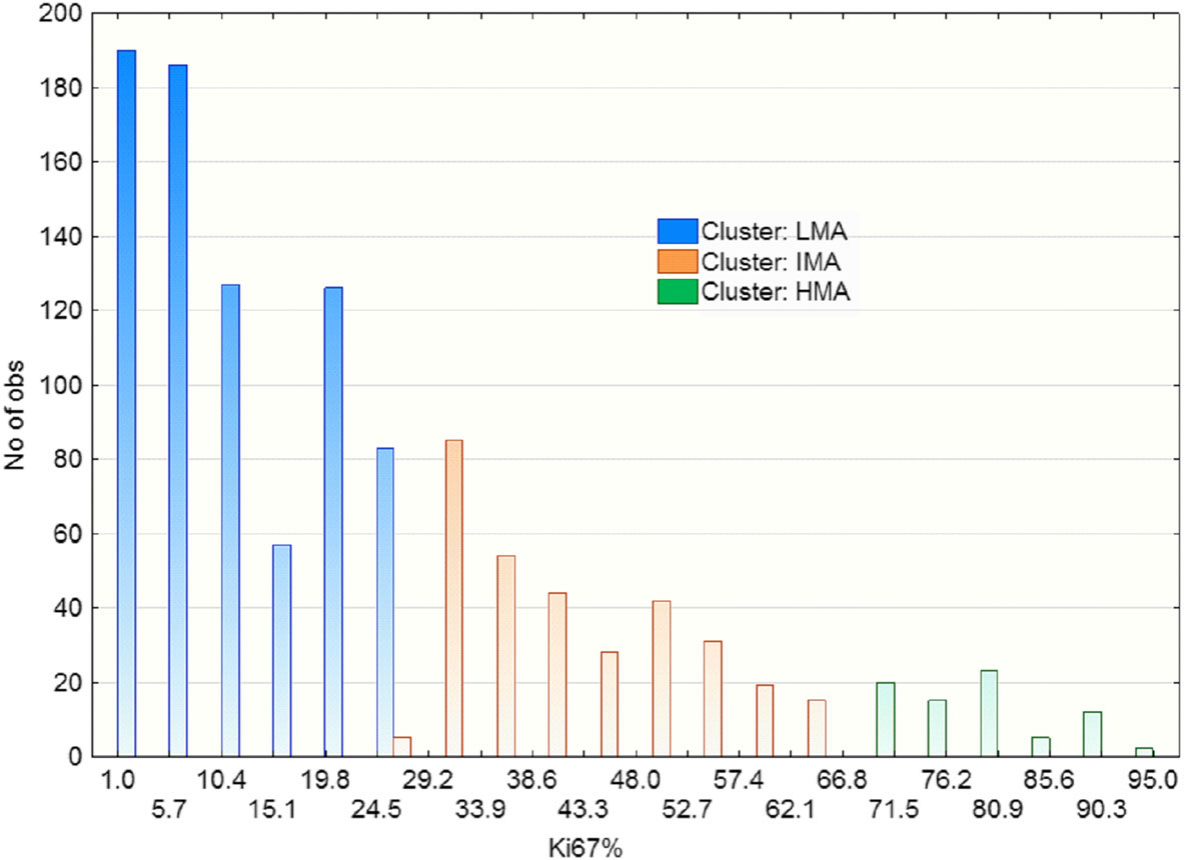
Histogram of three EM clusters in the pooled data of 1169 breast cancer patients (LMA - low mitotic activity; IMA - intermediate mitotic activity; HMA - high mitotic activity). These are the results of the second stage of EM clustering (details in Table 3)

To test these observations, data of all patients were pooled together and Ki67 values were tested for the presence of three EM clusters (here defined as pooled LMA, IMA and HMA clusters), shown in Table 3 and Fig. 3 with mean Ki-67 values (LMA 1.17%, IMA 40.45% and HMA 77.79%).

**Table 3:** Data of three EM clusters found in pooled data of 1169 breast cancer patients

Breast cancer patients	Three EM clusters from the pooled patients’ data						Values for all patients	
	LMA (low mitotic activity)		IMA (intermediate mitotic activity)		HMA (high mitotic activity)			
Mean Ki-67%	13.17		40.45		77.79		25.45	
St.dev. of Ki-67	8.43		13.77		8.45		21.08	
Breast cancer types	n	*%*	n	*%*	n	*%*	Total	*%*
Luminal A/B1	560	*80.92*	124	*17.92*	8	*1.16*	692	*100.00*
Luminal B2	126	*63.64*	68	*34.34*	4	*2.02*	198	*100.00*
triple-negative	41	*26.62*	58	*37.66*	55	*35.71*	154	*100.00*
pure HER2	42	*33.60*	73	*58.40*	10	*8.00*	125	*100.00*
Total	769	*65.78*	323	*27.63*	77	*6.59*	1169	*100.00*

These are the results of the second stage of EM clustering that identified the three overall clusters of Ki-67 values (LMA - low mitotic activity; IMA - intermediate mitotic activity; HMA - high mitotic activity). These are the results of the second stage of EM clustering

Table 4 shows unexpected distribution of our patients according to their tumor type (Luminal A/B1, Luminal B2, triple-negative and pure HER2) and cluster participation. A very few patients with ER+ tumors have been classified as belonging to the overall HMA cluster (some of them were PgR+ and other PgR-). On the other hand, ER- patients were classified to belong to all three overall clusters (29.75% LMA tumors, 46.95% IMA and 23.30% HMA tumors), clearly suggesting that their distribution of Ki-67 values differs substantially from ER+ patients. Dark grey cells in Table 4. mark the fields in which observed frequencies were above the expected frequencies, while the light grey cells mark the opposite situation in which observed frequencies were below expectation.

- Among 799 ER + PgR+ patients distribution in clusters was HER2 dependent (*p* = 0.000243), due to increased number of IMA HER2 3+ tumors on the expense of LMA HER2 3+ tumors (52 IMA out of 162 HER2 3+ patients versus113 IMA out of 637 HER2 < 3+ patients).
- This was not found among ER + PgR- patients (*p* = 0.186968). Mitotic activity of ER + PgR- tumors seems unrelated to HER2 status, possibly due to the presence of “dysfunctional” ER that do not stimulate PgR expression.
- Among ER-PgR- patients, HER2 overexpression also increased number of IMA tumor, but by reducing the number of HMA tumors (*p* < 0.000001). Here, difference between HER2 absent (0+) and HER2 3+ patients was evident (10 HMA out of 125 HER2 3+ patients versus 42 HMA out of 103 HER2 0+ patients), while patients with HER2 1+ or 2+ tumors did not differ from the expected frequencies, suggesting that at least among ER negative tumors, the absence of HER2 might be as important as the HER2 overexpression.

**Table 4:**
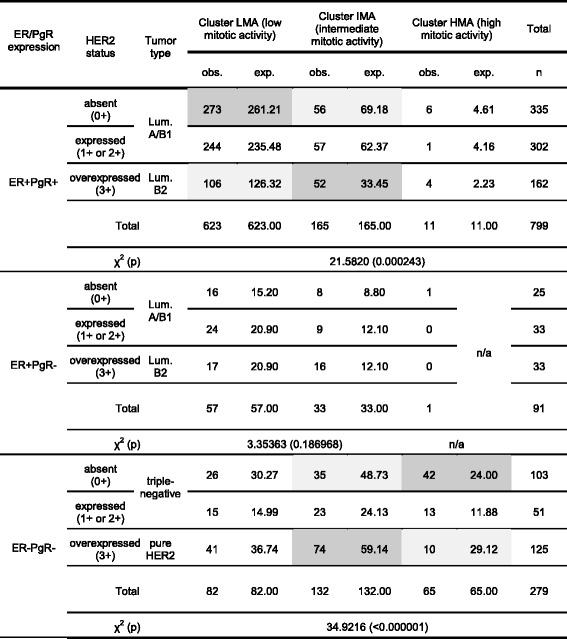
Distribution of breast cancer patients of a certain ER/PgR phenotype according to HER2 expression, tested by χ2 tests. These are the results of the second stage of EM clustering that identified the three overall clusters of Ki-67 values. Dark grey marks the fields in which observed frequencies were above the expected frequencies, while the light grey marks the opposite situation. HER2 overexpression in ER+PgR+ cancers increased the share of IMA tumors and reduced the share of LMA tumors (*p*=0.000243). Similar trends in ER+PgR- cancers were not significant (*p*=0.186968). Among ER-PgR- cancers, HER2 overexpression has reduced the share of HMA tumors, while increasing shares of other two clusters, particularly of IMA tumors (*p*<0.000001)

Taken all together, these results suggest that breast cancers can be divided in three levels of mitotic activity, with different mechanisms behind ER positive and ER negative tumors. In the former HER2 overexpression increases number of IMA tumors on the expense of LMA tumors, while in the latter HER2 overexpression reduces number of HMA tumors. A possible interpretation is that ER + PgR+ and ER negative breast tumors are intrinsically so different that the HER2 overexpression reduces number of LMA ER + PgR+ tumors and HMA ER-PgR- tumors. This is supported by the observation that HER2 absent (0+) tumors show highest shares of LMA ER + PgR+ and of HMA ER-PgR- cancers.

**Fig. 4:**
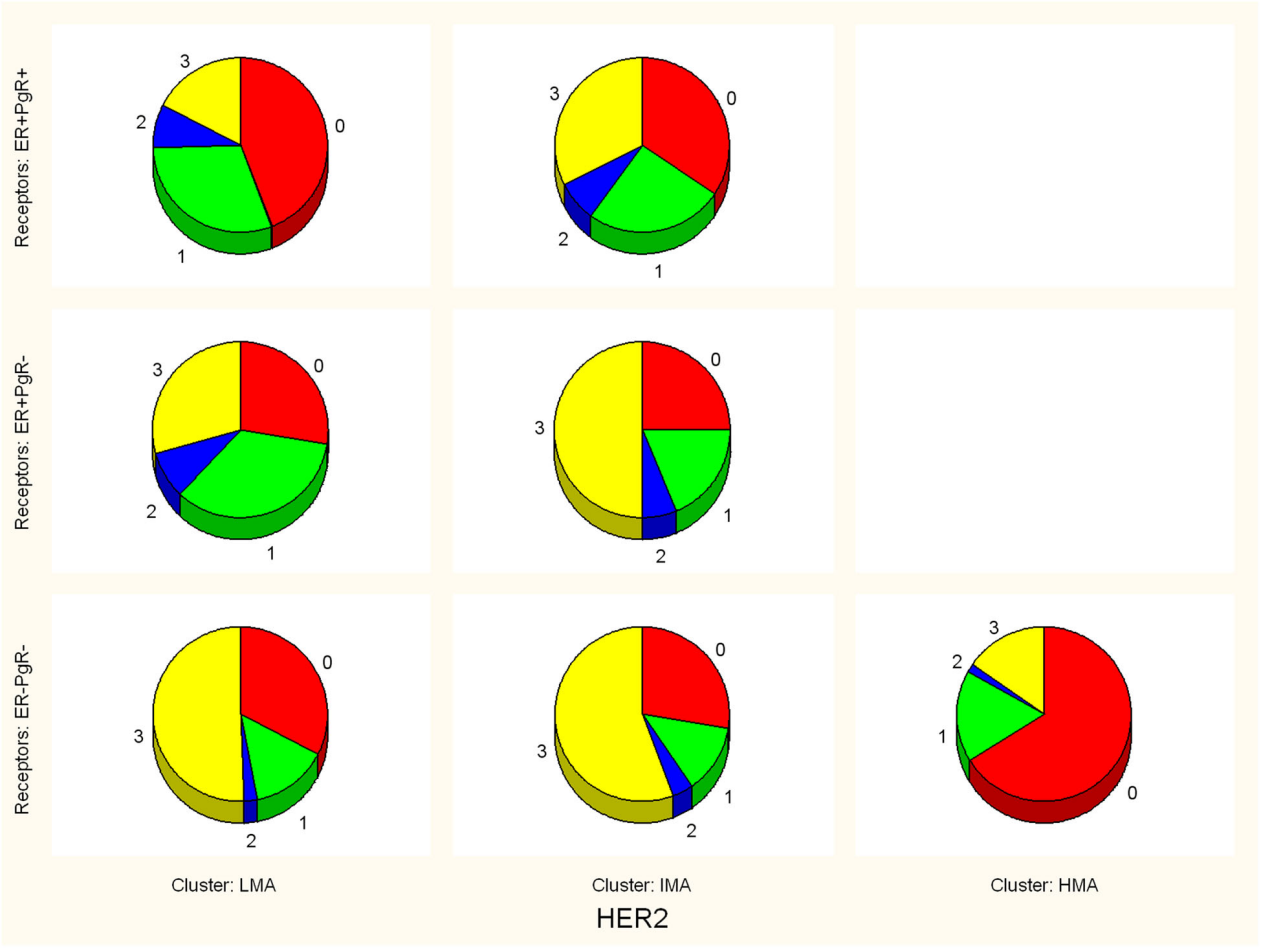
Pie charts of HER2 expression in three EM clusters of pooled breast cancer patients accordingly to their ER/PgR phenotype (LMA - low mitotic activity; IMA - intermediate mitotic activity; HMA - high mitotic activity). These are the results of the second stage of EM clustering (details in Table 4)

To make these observations more clear, distributions of the pooled HER2 expression data within the three clusters and three ER/PgR phenotypes are shown in Fig. 4. as seven pie charts.

## Discussion

### Possible promitotic mechanisms in breast tumor IHC phenotypes

Speed of the primary tumor growth mainly depends on the mitotic rate (routinely estimated by the Ki67 value) and on the rate of cancer cell destruction by apoptosis and other mechanisms that threaten the survival of tumor cells.

According to guidelines, breast cancer patients are after surgery treated according to their cancer type. Within Luminal tumors, Ki67 values define two cancer types, Luminal B1 and Luminal B2. This means that immunohistochemical phenotype of tumor tissue somehow influences the course of disease and effects of various treatments including targeted drugs. Here reported disparities in Ki-67 values between tumors with normally expressed HER2 (subset of patients with cancers expressing HER2 from 0+ to 2+) suggest that five common types of breast cancer are not as homogeneous as it can be expected.

Table 5 shows an attempt to interpret here presented relations between the phenotype variants and breast cancer biology among our patients. Here proposed explanation is that subgroups of breast cancer phenotypes differ in their Ki-67 distributions due to separate mechanisms that also include Ki-67 dependency on HER2 expression:

- Ki-67 values of tumors with functional ER (ER + PgR+ phenotype) seem dependent both on estrogen exposure and on the status of HER2 expression
- LMA & IMA clusters of PgR negative phenotypes (ER + PgR- and ER-PgR-) seem similar in their distributions of HER2 values, so HER2 is an unlikely candidate to explain increased Ki-67 values in IMA clusters of these two phenotypes, suggesting that some unknown promitotic mechanism might be involved.
- Tumors lacking both ER and PgR with high Ki-67 values (HMA clusters with values >65%) seem independent both of estrogen exposure and HER2 expression, so other promitotic mechanisms should be considered.

**Table 5:** The proposed interpretation of possible mechanisms behind distribution of Ki-67 values among subgroups of different immunohistochemical cancer phenotypes

HER2 & ER/PgR Breast cancer phenotypes		Model proposed subdivision of breast cancers, based on mitotic activity		
		LMA (low mitotic activity) Ki67 < 25%	IMA (intermediate mitotic activity)Ki-67 25–65%	HMA (high mitotic activity)Ki-67 > 65%
HER2 0+ to 3+	ER + PgR+	~67% of all patients probably HER2 dependent mitotic rates, thus intermediate mitotic rates seem dependent on the increased HER2 expression		~ 1% of all patients high mitotic rate due to unknown promiotic mechanism
	ER + PgR-	~26% of all patients mitotic rates do not seem closely regulated by normal HER2 expression (0+ to 2+), HER2 3+ increases number of IMA tumors		
	ER-PgR-			~6% of all patients high mitotic rate due to unknown promiotic mechanism, HER2 3+ reduces number of HMA tumors
Open questions		168 out of 769 LMA cancers were HER2 3+ Can HER2 molecules in HER2 3+ & LMA cancers be dysfunctional?	99 out of 323 IMA cancers were HER2 absent Is there another promitotic mechanism in HER2 absent & IMA tumors, particularly in 55 ER + PgR+ cancers?	43 out of 106 triple-negative & HER2 absent cancers were HMA What promotes the highest mitotic rates in HER2 absent ER-PgR- tumors?

A study by Wang XZ et al. [17] can be used as an illustration that less recognized tumor growth mechanisms have been proposed in triple-negative breast cancer patients. They have analyzed 264 patients with breast cancer divided into four molecular types plus the expression of p53 and EGFR. Triple-negative and HER2 overexpressed cancers were found to be larger and with higher Ki-67 as compared with the Luminal types. Beside that, triple-negative tumors showed less positive lymph nodes and higher CK5/6 and EGFR expression than the other three types, while p53 expression positively correlated with the EGFR expression only among triple-negative tumors, suggesting that tumor growth mechanism in triple-negative might differ from other breast cancers [17].

In triple-negative tumors promitotic mechanisms can include various mediators that do not interact with ER and PgR. Beside androgen receptor, EGFR ligands, activin/inhibin interactions also seem plausible [18–20].

Based on these observations, Table 5 also addresses few open questions regarding Immunohistochemical phenotypes of tumors of the three clusters based on their mitotic activity:

- If 168 cases out of our 769 breast cancers in the LMA cluster were HER2 3+, does this suggest that in these tumors HER2 molecules might be dysfunctional and thus result in unexpectedly low Ki-67 values despite the HER2 overexpression?
- If 99 out of our 323 breast cancers in the IMA cluster were HER2 absent, does this suggest that another promitotic mechanism should be searched for in HER2 absent & IMA tumors, particularly in those 55 cancers showing the ER + PgR+ phenotype?
- If 43 out of 106 our triple-negative & HER2 absent cancers belonged to the HMA cluster, is there some special feature that promotes the highest mitotic rates in triple-negative breast cancers with no HER2 molecules? It almost seems that among triple-negative tumors any status of HER2 presence is associated with a reduction in Ki-67 values.

### Progesterone receptor and breast cancer biology

The prevailing interpretation of the breast cancer occurrence is that increased or prolonged estrogen exposure leads to an increased risk for the development of breast cancer [21]. Estrogen via ER molecules stimulates proliferation of breast cancer cells and regulates the expression of other proteins in the tumor cells, including the progesterone receptor [22]. The presence of ER or PgR on breast cancer cells typically suggests slower-growing tumors, amenable to hormonal manipulation [23].

Here presented results suggest that the PgR expression on breast cancer cells is related to the Ki-67 value, here used as marker of tumor biology. It is important to note that the role of PgR expression in breast cancer cells remains not fully elucidated, since PgR expression is influenced by the estrogen milieu [7] and it has been reported that the lack of PgR in ER+ tumors is associated with worse survival [6]. A research study involving 327 ER+ breast cancer patients as shown that the Luminal B patients with PgR- tumors had a relatively higher pathological complete response rate than patients with PgR+ tumors (29.5% versus 4.7% pCR, *P* < 0.001), but in Luminal B patients with a residual tumor after neoadjuvant chemotherapy, PgR absence was independently correlated with poor relapse-free survival (*P* = 0.017) and overall survival (*P* = 0.013) [24]. These authors have concluded that the lack of PgR expression might be an important determinant of tumor biology in Luminal types of breast cancers.

Among 4115 patients with ER or PgR positive and not HER2 overexpressed breast cancers, reduced cancer-free intervals were noted in patients whose tumors had lower PgR expression and higher Ki-67 value [25]. This is possibly related to the second report that among 398 patients early relapses in patients with Luminal B and HER2-negative breast cancers were related to PgR negativity [26].

It remains unsettled whether the PgR expression threshold should be as low as 1% or higher. Among 1522 patients with primary breast cancer ER+/PgR−/HER2- tumors showed poorer clinicopathologic characteristics compared with ER+/PgR+/HER2- tumors using a PgR threshold of 20% instead of 1% [27]. In another report of 327 surgically removed ER positive and HER2 not-overexpressed breast cancers, only among postmenopausal patients it was reported that high Ki-67 value and low PgR expression (<20%) were significant independent factors for worse distant relapse-free survival [7].

## Conclusions

A plausible interpretation of our results is that the inherent mitotic activity of ER + PgR+ cancers is low and HER2 overexpression can act as a promitotic factor in some of these tumors showing the intermediate level of mitotic activity. It remains possible that the PgR expression also plays an important role here, without PgR, the link between the HER2 status and Ki-67 values vanishes. On the other hand, ER negative tumors seem to be inherently of very high mitotic activity, best evident in HER2 absent tumors, suggesting that any HER2 expression in ER negative cancers reduces mitotic activity to some extent. This means that at least among ER negative breast tumors, HER2 absence (0+) should be validated as a potential prognostic IHC feature.

Published reports open a question whether the altered biology of ER + PgR- breast cancers results from dysfunctional ER molecules (unable to promote the PgR expression) [28–32]. A less obvious alternative is that these tumors have normal ER, but due to some ER unrelated defect, cannot express PgR. In this case, the lack of PgR ligands’ actions on tumor cells without PgR might be the cause of altered Ki-67 values. The answer to this dilemma requires better understanding of interactions between nuclear and membrane receptors for estrogen and progesterone.

Nevertheless, the potential impact of here presented findings regarding PgR expression and Ki-67 values on patients’ management warrants a large prospective study of DFS and overall survival among breast cancer patients with various ER/PgR cancer phenotypes.

## Additional file

Additional file 1: All data analysed during this study are included in this published article and its supplementary information file: Breast_Cancer_HRV.xls that contains these variables: Tumor size (in cm); ER (0 or 1); PgR (0 or 1); HER2 (0 to 3); HER2_trinary (0, 1+ to 2+, 3+); Ki67% (in %); Tumor_Tyoe (A, B1, B2, 3 N, H);EM_Cluster (1 to 3). (XLS 125 kb)

## Abbreviations

EM clustering: Expectation maximization clustering
ER: Estrogen receptor
HER2: Human epidermal growth factor receptor 2
HMA: High mitotic activity
IMA: Intermediate mitotic activity
LMA: Low mitotic activity
PgR: Progesterone receptor
